# Functional Analysis of the Chemosensory Protein MsepCSP8 From the Oriental Armyworm *Mythimna separata*

**DOI:** 10.3389/fphys.2018.00872

**Published:** 2018-07-12

**Authors:** Aneela Younas, Muhammad I. Waris, Muhammad Tahir ul Qamar, Muhammad Shaaban, Sean M. Prager, Man-Qun Wang

**Affiliations:** ^1^College of Plant Science and Technology, Huazhong Agricultural University, Wuhan, China; ^2^College of Informatics, Huazhong Agricultural University, Wuhan, China; ^3^College of Resources and Environment, Huazhong Agricultural University, Wuhan, China; ^4^Department of Plant Sciences, University of Saskatchewan, Saskatoon, SK, Canada

**Keywords:** chemosensory protein, fluorescence competitive binding assay, molecular docking, behavioral response, RNAi

## Abstract

Chemosensory proteins (CSPs) play important roles in chemosensation in insects, but their exact physiological functions remain elusive. In order to investigate the functions of CSPs in the oriental armyworm *Mythimna separata*, in the present study we explored expression patterns and binding characteristics of the CSP, MsepCSP8. The distinctive functions of MsepCSP8 were also validated by RNAi. The results showed that MsepCSP8 shares high sequence similarity with CSPs of other insect family members, including the characteristic four-cysteine signature motif. MsepCSP8 mRNA was specifically expressed in antennae of females at levels well above those in other tissues. Competitive binding assays confirmed that 20 out of 56 ligands bound more strongly to MsepCSP8 at pH 7.4 than at pH 5.0. Protein structure modeling and molecular docking analyses identified amino acid residues involved in binding volatile compounds, and behavioral response experiments showed that *M. separata* elicited significant responses to five volatiles from compounds displaying high binding affinity to MsepCSP8. MsepCSP8 transcript abundance was decreased by dsMsepCSP8 injection, which affected the behavioral responses of *M. separata* to representative semiochemicals. Our findings demonstrate that MsepCSP8 likely contributes to mediating responses of *M. separata* adults to plant volatiles.

## Introduction

Olfaction is essential for identifying and analyzing volatiles in the environment (Carraher et al., 2015; Gadenne et al., 2016). Insects depend mainly on the peripheral olfactory system to recognize special semiochemicals in complex ecological habitats, which is important for foraging, host searching, mating, oviposition and other important physiological processes (Wang et al., 2015; Antony et al., 2016). Insects can detect and discriminate semiochemicals through numerous olfactory proteins, including chemosensory proteins (CSPs), odorant binding proteins (OBPs), odorant receptors (ORs), sensory neuron membrane proteins and odorant degrading enzymes (Kaupp, 2010; Leal, 2013).

Chemoreception is initiated in insects when chemicals bind to chemosensory-related proteins and are transported through the aqueous haemolymph (Brito et al., 2016; Pelosi et al., 2018). Exogenous volatiles, which are hydrophobic, must be solubilized before reaching the dendritic membranes of sensory neurons. OBPs and CSPs are believed to function during the initial stages of chemoreception in insects (Pelosi et al., 2018). Expression patterns of many OBPs have been observed in chemosensory tissues such as the olfactory sensilla (Biasio et al., 2015), whereas CSPs are found ubiquitously in olfactory and contact lymph sensilla in numerous insect species (Jin et al., 2005; Sun et al., 2014).

Chemosensory proteins are small, soluble, acidic, and generally comprised of 100-115 amino acids (Campanacci et al., 2003). CSPs possess four conserved cysteines that form two disulphide bridges. CSPs are compact polypeptides containing six α-helices that form a hydrophobic binding cavity (Tegoni et al., 2004). Phylogenetic analysis of CSPs from different insect species shows that CSPs are highly conserved and possess a characteristic N-terminal signature motif (Wanner et al., 2004; Forêt et al., 2007). CSPs perceive and bind external ligands, which result in action potentials that contribute to subsequent behaviors (Tunstall and Warr, 2012; He Y. et al., 2017). Previous studies have shown that CSPs are exclusively expressed in the antennae of *Sesamia inferens* (Zhang et al., 2014), *Polistes dominulus* (Calvello et al., 2003), *Linepithema humile* (Ishida et al., 2002), *Cerapachys biroi* (Mckenzie et al., 2014) and other insect species. CSPs have also been found in non-sensory tissues (Jin et al., 2006) suggesting they might be involved in insect growth and development (Liu Z. et al., 2016). However, their exact physiological functions and mechanisms remain elusive (Pelosi et al., 2018).

The oriental armyworm *Mythimna separata* (Lepidoptera: Noctuidae) is an overwhelming migratory and polyphagous pest of numerous cereal crops including rice, wheat and maize. It is widely distributed in Asia, Oceania, and Africa, where it affects both the quality and quantity of crop yields (He P. et al., 2017). *M. separata* migrates to long distances even up to 1000 km per season (Liu et al., 2017). The larvae of *M. separata* feed on plants, and completion of their life cycle causes serious economic losses. Recent outbreaks of *M. separata* have been observed in several regions of China, especially Jilin, Hebei, Heilongjiang, Liaoning, and Shanxi, and this organism poses a severe threat to corn production (Yun et al., 2017). In an attempt to restrict crop damage by *M. separata*, high doses of synthetic insecticides are often applied (Liu Y. et al., 2016), but this causes serious environmental problems and cropland deterioration, stimulates insect resistance, and has a negative impact on non-target organisms (Younas et al., 2016). Population outbreaks of *M. separata* present a great challenge in terms of protecting crops worldwide (Liu et al., 2017).

In order to better control *M. separata*, novel management tactics and molecular insight into olfactory mechanisms are needed, but only a few studies on olfactory-related genes in *M. separata* have been conducted, and functional characterization of CSPs has not been reported. In our previous study, several CSPs were identified in *M. separata* from antennae transcriptome analysis (Chang et al., 2017). In order to explore the functions of CSPs, we evaluated expression profiles of the *M. separata* CSP gene MsepCSP8, analyzed ligand binding affinity and structural properties by molecular docking, and performed targeted gene silencing using RNAi combined with behavior bioassays.

## Materials and Methods

### Insects and Collection of Tissues

*Mythimna separata* were provided by Hubei Academy of Agricultural Sciences, Wuhan, China, and maintained under controlled conditions (temperature of 25 ± 1°C and relative humidity of 70% ± 5%). Male and female adults were kept in separate cages and fed on 10% honey solution. Larvae, pupae and adults (whole body), and antennae, head without antennae, thorax, abdomen, wings and legs tissues from both sexes were collected (three replicates) for RT-qPCR analyses. Samples were stored at –80°C until further use.

### RNA Extraction and cDNA Synthesis

To analyze the abundance of MsepCSP8 transcripts, total RNA was extracted from larvae (6th instar), pupae (5 days old), the whole bodies (3 days old), and body tissues (antennae, head, thorax, abdomen, legs and wings) of both sexes (1–5-days-old) using TRIzol reagent (Invitrogen, Carlsbad, CA, United States). To investigate the efficacy of RNAi on MsepCSP8 expression levels, total RNA was extracted from whole bodies at 1, 2, 3, 4, and 5 days post-eclosion from non-injected controls, dsGFP- and dsRNA-injected male and female adults. The purity of RNA was quantified by agarose gel electrophoresis and the concentration was determined using a spectrophotometer (Nanodrop-2000, Massachusetts, United States). The first-strand cDNA for RT-PCR and RT-qPCR were synthesized from 1 μg of total RNA using MBI RevertAid First Strand cDNA kit (MBI Fermentas, Glen Burnie, MD, United States) and PrimerScript RT Reagent kits with gDNA Eraser (Perfect Real Time; Takara), respectively, according to manufacturer’s instructions, and stored at –20°C until further use.

### Phylogenetic Analysis

The similarity of MsepCSP8 to various homologs from different insect orders was analyzed using NCBI BLAST^1^. All amino acid sequences, including MsepCSP8, were aligned by ClustalW 1.83 (Thompson et al., 1997). A neighbor-joining phylogenetic tree was constructed based on amino acid sequences using MEGA6 (Tamura et al., 2013). Node support was assessed using a bootstrap procedure with 1000 replicates, and uniform rates with pairwise deletion of data gaps.

### Real-Time Quantitative PCR

MsepCSP8 expression patterns were analyzed by RT-qPCR using a Bio-Rad Real-time thermal cycler CFX96 detection system (Applied Biosystems, United States). For RT-qPCR analysis, primers were designed (**Supplementary Table S1**) based on sequences from the NCBI database^2^. Each RT-qPCR sample contained 10 μl of 2 × SYBR Green qPCR Mixture (Aidlab, China), 1 μl of 0.1 μg/10 μl cDNA, 0.5 μl of gene-specific primers (from 10 μM stock solution) and 8 μl of sterilized ultrapure water. RT-qPCR was conducted at 95°C for 3 min, followed by 40 cycles at 95°C for 10 s and 55°C for 30 s. MsepCSP8 gene expression was normalized against the β-actin gene of *M. separata* (GQ856238). In order to validate the efficiency of PCR amplification, each primer was analyzed following serial dilution (at least five orders of magnitude) of template (three replicates), and the resultant efficiency was >90%. To obtain optimal results, each sample was assessed with three technical and three biological replicates. To differentiate mRNA expression levels in *M. separata*, the comparative 2^-ΔΔ^*^C^*^T^ method was performed as described by Livak and Schmittgen (2001). One-way analysis of variance (ANOVA) was performed to identify significant differences in expression levels in all tested samples using SPSS version 16.0 for Windows. Differences were evaluated at *p*≤ 0.05.

### Construction of Recombinant Plasmid

Amplification of MsepCSP8 was carried out by PCR with a forward primer (5^′^-CCGGAATTCATGAAAACCTTATTCA-3^′^) containing an *EcoR*I restriction site and a reverse primer (5^′^-CCGCTCGAGTTATTGAGAGACTTCTT-3^′^) containing an *Xho*I restriction site. The PCR product was ligated with the pTOPO-T vector (Aidlab), and the resulting construct was transformed into competent *Escherichia coli* DH5α cells. Positive clones were grown in Luria-Bertani (LB) medium with kanamycin (50 μg ml^-1^) and subsequently sequenced. The target fragment was digested with *EcoR*I and *Xho*I restriction enzymes and ligated into the pET-30a plasmid. The recombinant plasmid (purified using Omega Plasmid Mini Kit-I) was transformed into competent *E. coli* DH5α cells, and after DNA sequencing, competent *E. coli* BL21 (DE3) cells were transformed with the confirmed recombinant plasmid. A single clone was grown in LB medium containing 50 μg ml^-1^ of kanamycin with shaking at 220 rpm on a mechanical shaker for ∼12 h at 37°C, and then sequenced.

### Expression and Purification of Recombinant Protein

Positive colonies were confirmed by DNA sequencing. Expression and purification was conducted using transformed cells grown in LB medium (5 ml) with kanamycin (50 μg ml^-1^) with shaking at 220 rpm at a temperature of 37°C for 12 h. Cultures were diluted to 1000 ml with LB medium and grown to an OD_600_ value of 0.4–0.6. To induce protein expression, 0.1 mmol L^-1^ isopropyl-beta D-thiogalactopyranoside (IPTG) was added and culturing continued for 4 h at 37°C.

The expressed protein was obtained in soluble form in the supernatant, and purification was accomplished using a Ni affinity chromatography column (GE Healthcare, Uppsala, Sweden). Bound protein was eluted by digestion with recombinant bovine enterokinase at 26°C for 15 h to remove the His-tag. Expression and purification were verified by 15% sodium dodecyl sulfate-polyacrylamide gel electrophoresis (SDS-PAGE), and protein concentration was measured as described by Cao et al. (2017). Dialysis of purified protein was carried out in 30 mM Tris-HCl buffer [Tris(hydroxymethyl)aminomethane] at pH 7.4 and pH 5.0 before fluorescence binding assays, and protein was stored at -80°C until use.

### Fluorescence Binding Assay

Fluorescence competitive binding assays were performed to determine the binding affinity of MsepCSP8 for 56 ligands using *N*-phenyl-1-naphthylamine (1-NPN) as a fluorescent probe. Stock solutions of ligands were prepared in spectrophotometric-grade methanol. To test the binding affinity, protein (1 mM) was diluted with Tris-HCl buffer (30 mM) to a final concentration of 20 μmol l^-1^, and this was mixed with 1-NPN (1 mM) to achieve a final concentration of 0–20 μmol l^-1^. Mixtures of 1-NPN and protein were excited at 337 nm wavelength, and emission spectra were recorded from 360 to 600 nm using a fluorescence spectrophotometer (RF-5301PC Shimadzu, Kyoto, Japan). Binding of selected ligands with MsepCSP8 (1:1 protein:ligand stoichiometry) was evaluated using 1-NPN as a fluorescent probe, with the final concentration of the product ranging from 0 to 20 μM. A decline in the fluorescence intensity of the ligand indicates that 1-NPN bound to MsepCSP8 was displaced by the ligand. Three independent measurements were made for all experiments. The binding affinity (Ki) of MsepCSP8 for all tested ligands was calculated based on IC_50_ values as follows:

$$\mathrm{Ki}=IC_{50}/(1\;+\;\left[1-\mathrm{NPN}]/K1–\mathrm{NPN}\right)$$

where [1-NPN] is the free concentration of 1-NPN, and K1-NPN is the dissociation constant of the complex of MsepCSP8 and 1-NPN.

### Modeling of Three-Dimensional (3D) Structure and Molecular Docking of Ligands

A Delta-BLAST search was performed against the NCBI database^3^ and the MsepCSP8 amino acid sequence was searched against the protein data bank (PDB^4^) using SWISS-MODEL^5^. The sequences resulting from BLAST searches with >60% similarity were subjected to multiple alignment using ClustalW2. The top hit was selected based on query coverage, number of cysteine residues, phylogeny and sequence homology. The structure of CSPsg4 from *Schistocerca gregaria* (accession no. 2GVS_A) was used as template to construct a 3D model of MsepCSP8 (Tomaselli et al., 2006). We used the docking protocol implemented in MOE (version 2012.10) designed by Chemical Computing Group (Vilar et al., 2008) for molecular docking. For further prediction of MsepCSP8 binding sites, ligands (hexanal, terpinolene, 2-tridecanone, α-terpinene, 1-penten-3-ol, cyclohexanol, octanal, *trans*-2-hexenal and [-]-terpinen-4-ol) exhibiting strong binding affinities with MsepCSP8 were docked into the binding pocket of the model. Specific parameters used for calculation of interactions between ligands, and scores for corresponding ligands using default parameters, were rescoring-1 (London-dG, Refinement: Force-field) and rescoring-2 (GBVI/WSA-dG, Placement: Triangle-Matcher). The optimal structure was selected based on root mean square deviation (RMSD) values and minimum *S*-score. *S*-score denotes the calculated value from the built-in scoring function of MOE based on the ligand binding affinity to the receptor protein after docking. The RMSD value is used for comparing docked conformations with reference conformations/other docked conformations. Ligands with low RMSD values and minimum S-scores are considered to have the potential to be developed as potential inhibitors (Qamar et al., 2016).

### Double-Stranded RNA Synthesis

The MsepCSP8 cDNA was sub-cloned into the pTOPO-T vector and diluted construct was used as template for amplification of the target sequence. The MsepCSP8 sequence was amplified by PCR using specific primers conjugated with 19 bases of the T7 RNA polymerase promoter (**Supplementary Table S1**). The 378 bp MsepCSP8 and 460 bp GFP PCR products were purified and used as templates for dsRNA synthesis using a Promega kit (Beijing, China) according to the manufacturer’s instructions. The synthesized dsRNA was precipitated with isopropanol, resuspended in nuclease-free water, and quantified by a spectrophotometer (Thermo Scientific, Nanodrop-2000, Wilmington, DE, United States). The purity and integrity of dsRNA was confirmed by 1% agarose gel electrophoresis and stored at -80°C until use.

### Double-Stranded RNA Injection and Gene Expression Analysis

The dsRNA (500 nl of 10 ng nl^-1^) was injected into 3rd and 4th abdominal segment of 7-day old pupae using a microinjector (World Precision Instruments Inc., Sarasota, FL, United States) under a microscope. After injection, pupae were kept in petri dishes until eclosion at 25 ± 1°C and 70 ± 5% relative humidity. Three treatments were set up for RNAi consisting of non-injected (controls), dsGFP-injected and dsRNA-injected (dsMsepCSP8) groups. After eclosion, adults from each treatment were kept in separate containers. Three individuals of each sex from all three treatments were taken at 1, 2, 3, 4, and 5 days post-eclosion for RNAi analysis. RT-qPCR was performed using the method described above.

### Olfactometer Bioassay

*Mythimna separata* behavioral responses were examined by olfactometer bioassays (Cao et al., 2015) performed in a glass Y-tube (base = 4.0 cm diameter by 25 cm length, arms = 3.0 cm diameter by 26 cm length) using 10 different volatile compounds (Sigma-Aldrich).

Air entering the tube was passed through activated charcoal for filtration and humidified using deionised water. Filtered air was split between two chambers; one chamber containing liquid paraffin (used as control) and the other containing the test volatile. Each chamber was connected to one arm of the Y-tube. Airflow was kept constant at 6.0 l min^-1^ throughout the experiment using an inline flow meter (Gilmont Instr., Barnant Co., Barrington, IL, United States). Orange light was used for this experiment. The whole experiment was conducted in a dark room, and the relative humidity and temperature were maintained at 70 ± 5% and 25 ± 1°C, respectively. A 10 μl of volatile compound was applied to 10 mm × 10 mm filter papers (Whatman No. 1) that was placed in the chamber connected to one arm of the Y-tube. A separate Y-tube was used for male and female moths.

To perform bioassays, a 3-day-old moth, either male or female, was released in the base of the Y-tube. Each insect was given 10 min to respond to the treatment, and the number of moths showing attraction, repulsion or no response was counted. Approximately 30 min before onset of the trial, male and female adults were placed into separate glass tubes (50 ml) and covered with a cotton plug to avoid exposure to tested volatiles. In total, 60 adults (30 male and 30 female) were tested per volatile compound, and χ^2^ tests were applied to evaluate the number of *M. separata* individuals attracted to or repelled by volatiles.

Y-tube bioassays were performed both before and after RNAi application to confirm the MsepCSP8 gene silencing effect. Three treatments consisting of dsGFP-injected, dsRNA-injected (dsMsepCSP8), and controls were tested on both 3-day old male and female moths. For post-RNAi bioassays, 30 male and 30 female *M. separata* individuals were examined using the volatile compounds terpinolene and hexanal. These were selected based on the observation of significant responses of *M. separata* to these compounds in bioassays conducted before injection of RNAi.

## Results

### Cloning and Phylogenetic Analysis of MsepCSP8

**FIGURE 1:**
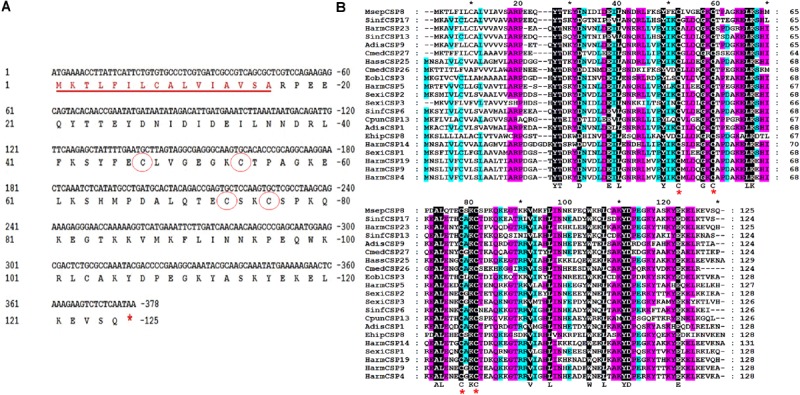
Cloning and phylogenetic analysis of MsepCSP8 from *Mythimna separata*. **(A)** Nucleotide and deduced amino acid sequence of MsepCSP8 from *M. separata*. The four conserved cysteines are displayed in red circle and stop codon is denoted by red asterisk. The underlined red sequence represent the sixteen amino acid predicted signal peptide. **(B)** Alignment of amino acid sequence of MsepCSP8 with other insect species. Four conserved cysteines are displayed with red star. The black, purple and sky blue color represent 100, 80, and 60% sequence similarity. The name and the GenBank accession no. of insect species are listed as follows: SinfCSP17, *Sesamia inferens*, AGY49266.1; HarmCSP23, *Helicoverpa armigera*, AIW65102.1; SinfCSP13, *Sesamia inferens*, AGY49262.1; AdisCSP9, *Athetis dissimilis*, AND82451.1; CmedCSP27, *Cnaphalocrocis medinalis*, ALT31609.1; HassCSP25, *Helicoverpa assulta*, ASA40086.1; CmedCSP26, *Cnaphalocrocis medinalis*, ALT31608.1; EoblCSP3, *Ectropis oblique*, ALS03828.1; HarmCSP5, *Helicoverpa armigera*, AEB54579.1; SexiCSP2, *Spodoptera exigua*, ABM67689.1; SexiCSP3, *Spodoptera exigua*, ABM67690.1; SinfCSP6, *Sesamia inferens*, AGY49255.1; CpunCSP13, *Conogethes punctiferalis*, APG32550.1; AdisCSP1, *Athetis dissimilis*, ALJ93810; EhipCSP8, *Eogystia hippophaecolus*, AOG12892.1; HarmCSP14, *Helicoverpa armigera*, AFR92098.1; SexiCSP1, *Spodoptera exigua*, ABM67688.1; HarmCSP19, *Helicoverpa armigera*, AIW65098.1; HarmCSP9, *Helicoverpa armigera*, AFR92093.1; HarmCSP4, *Helicoverpa armigera*, AEX07269.1.

The full-length cDNA encoding MsepCSP8 was cloned and verified by sequence analysis, revealing an open reading frame (ORF; GenBank Accession No. JAV45873.1) consisting of 378 nucleotides encoding a 125 amino acid polypeptide with a molecular weight of 14.40 kDa (**Figure 1A**). Analysis identified a signal peptide of 16 amino acid residues, and ExPASy^6^ predicted an isoelectric point of 7.52. Alignment of MsepCSP8 with homologous CSPs from other Lepidopteran species revealed the presence of the four conserved cysteines and sequence identity of 52–70% (**Figure 1B**). The results of BLASTx showed that MsepCSP8 shares relatively high amino acid similarity (>60%) with CSPs from other insect species in NCBI. A neighbor-joining tree was constructed based on amino acid sequences of 114 CSPs belonging to different insect orders including Lepidoptera (15 species), Hemiptera (12 species), Coleoptera (nine species), Diptera (five species), Hymenoptera (three species), Orthoptera (two species), Neuroptera (two species) and Dictyoptera (one species) (**Supplementary Figure S1**). The dendrogram revealed that MsepCSP8 shares a close ancestor from the same insect order.

### Tissue-Specific Expression of MsepCSP8

**FIGURE 2:**
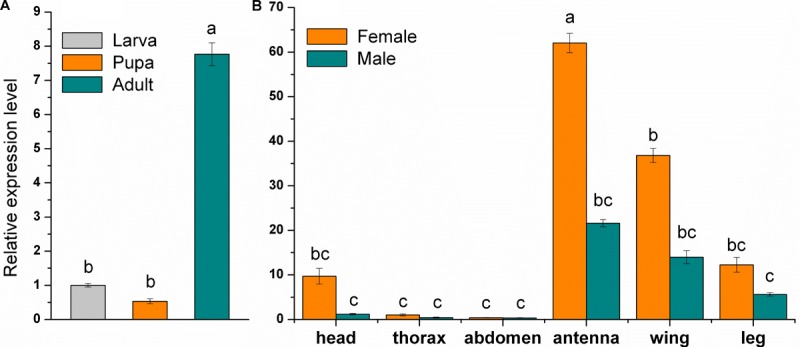
Relative expression level of MsepCSP8 from *M. separata*. **(A)** Expression level in larvae, pupae, and adult, **(B)** expression level in various tissues (head without antennae, thorax, abdomen, antennae, wings, and legs) of male and female *M. separata*. The different letters on each bar show significant differences evaluated at *p* ≤ 0.05. Each sample was tested three times as technical and three times as biological replicates (Mean ± SE).

Analysis of relative expression levels in larvae, pupae and adults, and in different tissues (head, thorax, abdomen, antennae, wings, and legs) of both sexes showed that MsepCSP8 has a broad expression profile in *M*. *separata*. MsepCSP8 expression was significantly (*p* < 0.05) higher in adults than in larvae and pupae (**Figure 2A**). Moreover, transcript levels were greater in females than males in all body tissues (**Figure 2B**). Notably, MsepCSP8 was expressed most highly in antennae, and expression levels were 2.9-fold higher in females than males. MsepCSP8 expression was also evident in wings of both females and males, and lower in legs than antennae and wings in both females and males. Thorax and abdomen tissue displayed minimal expression, and expression was markedly higher in the heads of females.

### Fluorescence Binding Assays

**FIGURE 3:**
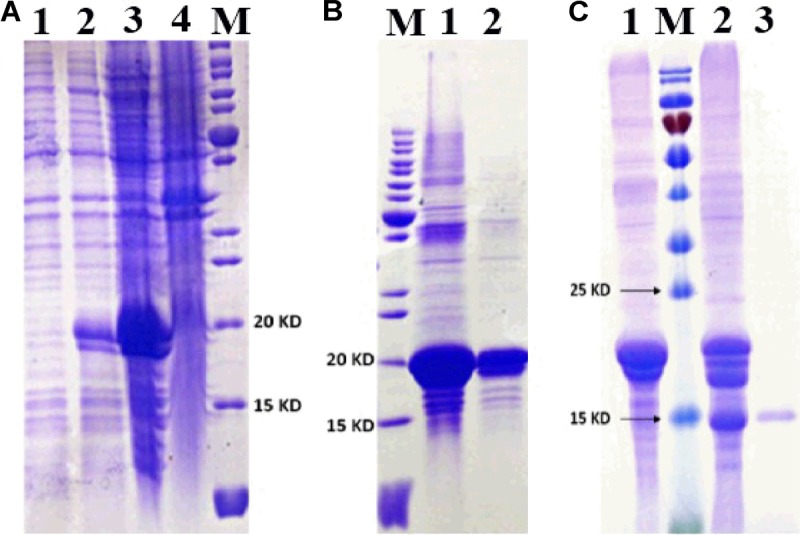
SDS-PAGE analyses displayed the expression and purification of MsepCSP8. **(A)** 1, non-induced MsepCSP8/pET30a; 2, induced MsepCSP8/pET30a; 3, induced MsepCSP8 lysate supernatant; 4, inclusion body; M, molecular marker, **(B)** M, molecular marker, 1 and 2, eluted protein before cleavage, **(C)** 1, eluted protein before cleavage; M, molecular marker; 2, digested with recombinant enterokinase; 3, purified protein cleaved by His-Tag.

**FIGURE 4:**
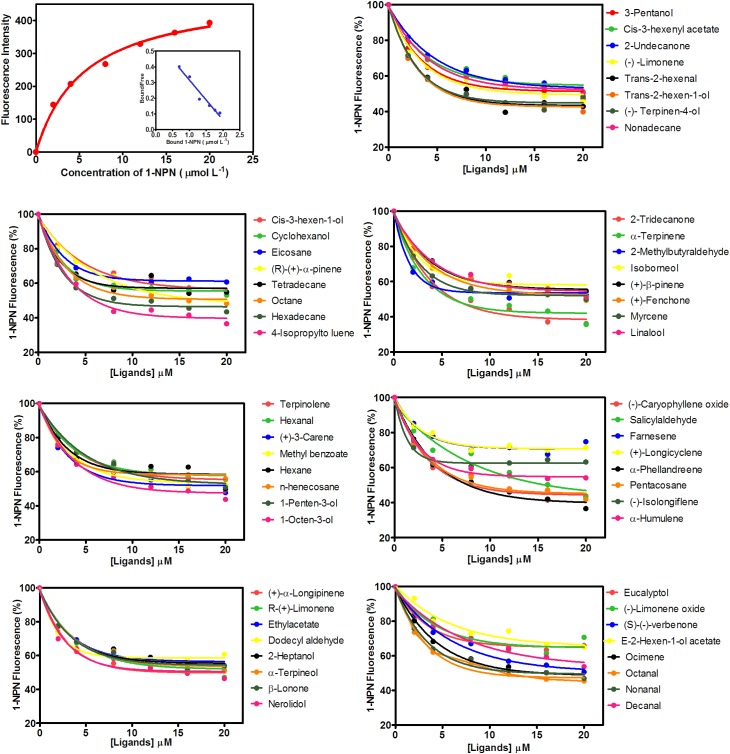
Competitive-binding assays of MsepCSP8 at pH 5.0. Binding curve of 1-NPN and relative Scatchard plot analysis (top left corner), and competitive binding curves of MsepCSP8 with different volatile groups (Alcohols, Esters and Benzoates, Ketone, Aldehydes, Alkane, Monoterpene, and Sesquiterpenes).

**FIGURE 5:**
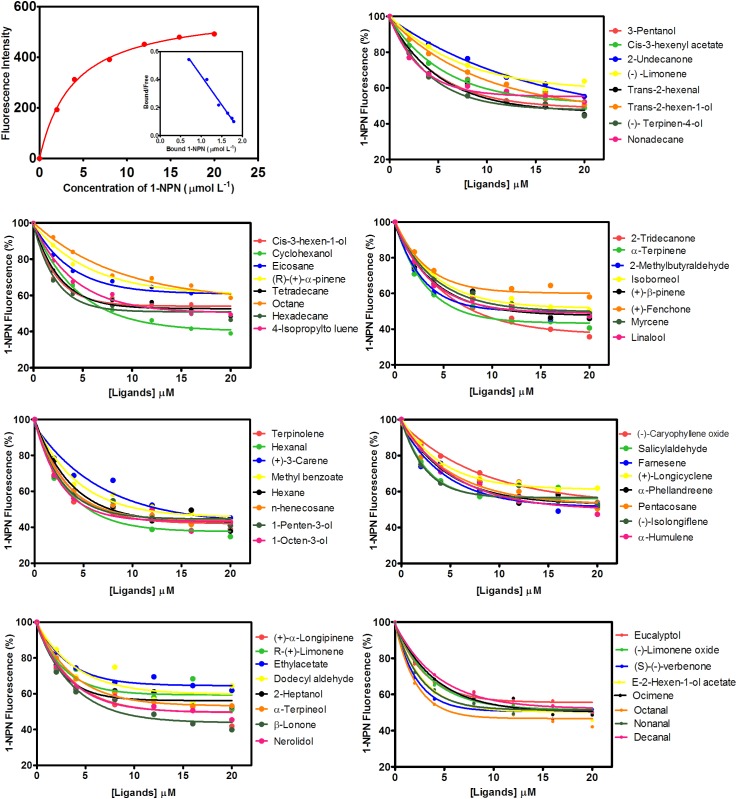
Competitive-binding assays of MsepCSP8 at pH 7.4. Binding curve of 1-NPN and relative Scatchard plot analysis (top left corner), and competitive binding curves of MsepCSP8 with different volatile groups (Alcohols, Esters and Benzoates, Ketone, Aldehydes, Alkane, Monoterpene, and Sesquiterpenes).

MsepCSP8 expression and purification was confirmed by 15% SDS-PAGE (**Figure 3**). The binding affinities of MsepCSP8 to various ligands were analyzed by fluorescence binding assays. Binding of the fluorescent probe *N*-phenyl-1-naphthylamine (1-NPN) with MsepCSP8 was analyzed and gradual saturation was observed. Saturation and linear Scatchard plots were constructed at pH 7.4 and pH 5.0, yielding dissociation constants of 2.41 and 2.93 μM, respectively (**Figures 4, 5**). Displacement curves of 1-NPN with various ligands are presented in **Figures 4, 5**, and IC50 and Ki values were calculated for all ligands (**Table 1**). Considering the influence of pH on the binding and release mechanisms of ligands to/from proteins, we performed experiments at pH 7.4 and pH 5.0 in order to simulate the pH-dependent environments in insect *in vitro*.

**Table 1:** Binding affinity of MsepCSP8 with different volatile groups (Alcohols, Esters and Benzoates, Ketone, Aldehydes, Alkane, Monoterpene and Sesquiterpenes).

Chemical name	CAS number	Purity (%)	pH 7.4		pH 5.0	
			IC50 (μM)	Ki (μM)	IC50 (μM)	Ki (μM)
**Alcohols**						
3-Pentanol	584-02-1	98	16.03	11.25	17.25	12.87
*Trans*-2-hexen-1-ol	928-95-0	96	23.76	16.67	8.46	6.31
*Cis*-3-hexen-1-ol	928-96-1	97	21.44	15.05	29.83	22.25
Cyclohexanol	108-93-0	99	9.58	6.72	21.72	16.20
1-Penten-3-ol	616-25-1	98	9.27	6.50	22.24	16.58
1-Octen-3-ol	3391-86-4	98	8.25	5.79	13.24	9.88
2-Heptanol	543-49-7	98	23.84	16.73	25.63	19.11
**Esters and benzoates**						
*Cis*-3-hexenyl acetate	3681-71-8	98	21.03	14.76	24.61	18.36
Methyl benzoate	93-58-3	99.5	12.54	8.80	21.16	15.78
Ethyl acetate	141-78-6	99.8	67.31	47.24	29.17	21.76
E-2-Hexen-1-ol acetate	3681-82-1		14.57	10.23	62.85	46.88
**Ketones**						
2-Undecanone	112-12-9	98	35.51	24.92	22.29	16.62
2-Tridecanone	593-08-8	98	9.07	6.37	7.75	5.78
β-Lonone	79-77-6	96	10.28	7.22	22.78	16.99
(+)-Fenchone	4695-62-9	99	29.69	20.84	20.02	14.93
**Aldehydes**						
*Trans*-2-hexenal	6728-26-3	97	12.54	8.80	8.85	6.60
Hexanal	66-25-1	95	6.75	4.74	32.66	24.36
Octanal	124-13-0	98	9.87	6.93	13.70	10.22
Nonanal	124-19-6	98	12.71	8.92	14.86	11.08
Decanal	112-31-2	97	20.45	14.35	31.05	23.16
Dodecyl aldehyde	112-54-9	92	24.61	17.27	36.85	27.48
Salicylaldehyde	90-02-8	99	21.32	14.97	16.29	12.15
2-Methylbutyraldehyde	96-17-3	95	13.76	9.66	21.43	15.98
**Alkanes**						
Nonadecane	629-92-5	99	25.05	17.58	19.82	14.79
Eicosane	112-95-8	99	48.01	33.70	46.34	34.57
Tetradecane	629-59-4	99	17.95	12.60	23.80	17.75
Octane	111-65-9	98	37.04	26.00	15.87	11.84
Hexadecane	544-76-3	99.8	14.82	10.40	10.26	7.65
Hexane	110-54-3	95	9.69	6.80	26.30	19.61
n-Heneicosane	629-94-7	99	9.59	6.73	36.50	27.22
Pentacosane	629-99-2	99.5	24.37	17.10	11.21	8.36
**Monoterpene**						
α-Terpineol	10482-56-1	90	20.57	14.44	21.88	16.32
(–)-Terpinen-4-ol	20126-76-5	95	13.88	9.74	9.46	7.06
(–)-Limonene	5989-54-8	95	40.43	28.38	15.08	11.25
R-(+)-Limonene	5989-27-5	95	23.53	16.52	18.85	14.06
(–)-Limonene oxide	1195-92-2	97	17.52	12.30	46.86	34.95
Myrcene	123-35-3	85	16.58	11.64	17.42	12.99
Linalool	78-70-6	97	14.46	10.15	25.03	18.67
(R)-(+)-α-pinene	7785-70-8	98	33.00	23.16	17.94	13.38
(R)-(+)-β-pinene	19902-08-0	98	13.86	9.73	26.37	19.67
(S)-(-)-verbenone	1196-01-6	97	13.21	9.27	21.52	16.05
α-Phellandrene	99-83-2	95	20.93	14.69	9.16	6.83
Isoborneol	124-76-5	95	19.08	13.39	32.30	24.09
(+)-3-Carene	13466-78-9	90	13.43	9.42	17.72	13.22
Ocimene	13877-91-3	95	16.72	11.73	15.58	11.62
Eucalyptol	470-82-	98	25.23	17.71	88.08	65.70
α-Terpinene	99-86-5	85	9.18	6.45	8.15	6.08
Terpinolene	586-62-9	85	8.82	6.19	27.01	20.15
**Sesquiterpenes**						
Farnesene	502-61-4	98	18.60	13.06	20.16	15.04
α-Humulene	6753-98-6	96	18.52	13.00	23.09	17.22
(–)-Caryophyllene oxide	1139-30-6	99	19.57	13.74	10.28	7.67
Nerolidol	7212-44-4	98	14.96	10.50	15.42	11.50
(+)-Longicyclene	1137-12-8	95	40.11	28.15	394.98	294.61
(–)-Isolongifolene	1135-66-6	98	24.62	17.28	61.63	45.97
(+)-α-longipinene	5989-08-2	99	14.05	9.86	14.41	10.75
4-Isopropyltoluene	99-87-6	98	17.99	12.63	7.72	5.76

A total of 56 plant volatiles consisting of alcohols, esters, benzoates, ketones, aldehydes, alkanes, monoterpenes, and sesquiterpenes were analyzed for binding to MsepCSP8 at pH 5.0 and pH 7.4. The pH significantly influenced the ligand binding, which was stronger at pH 7.4. Considering Ki < 10 μM as a standard value, 20 ligands displayed strong binding at pH 7.4, whereas most of the tested ligands showed comparatively low binding at pH 5.0.

### Structural Modeling and Molecular Docking

**FIGURE 6:**
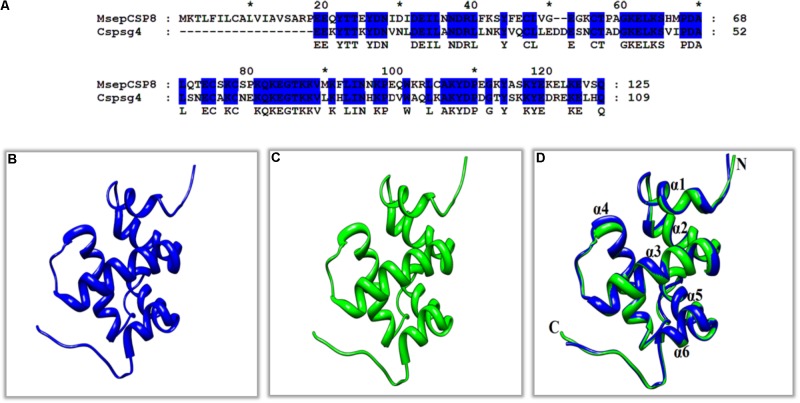
Three-dimensional (3D) structures of MsepCSP8. **(A)** Alignment of MsepCSP8 and CSPsg4 sequence, **(B)** 3D structure of MsepCSP8, **(C)** 3D structure of CSPsg4 used as a template, **(D)** Superimposed structure of MsepCSP8 and the template CSPsg4. Blue and green color indicate 3D structure of MsepCSP8 and template of CSPsg4 in the superimposed structure, respectively. C, C-terminal; N, N-terminal. The asterisk (^∗^) indicates the length of the amino acid.

The sequence of MsepCSP8 from *M. separata* was compared with previously characterized proteins in the Protein Data Bank (PDB). The results demonstrated that MsepCSP8 shares 61% similarity with CSPsg4 from *S. gregaria*, and the 3D structure of MsepCSP8 was modeled based on that of CSPsg4 as a template (**Figures 6A,B**). From the results of homology modeling, the best model (**Figure 6C**) was selected based on root mean square deviation (RMSD) value (0.386 Å) and its quality was confirmed by a Ramachandran plot of φ and ψ values (**Supplementary Figure S2**), which revealed 96% of the residues in favored regions. The plot also indicated that a high proportion of residues were in α-helices, as expected. The predicted structure demonstrated that MsepCSP8 is an α-helix-rich globular protein consisting of six α-helices; α-1 (Ile31-Asn36), α-2 (Asp38-Val49), α-3 (Gly58-Gln70), α-4 (Pro78-Asn94), α-5 (Pro96-Tyr106), and α-6 (Ala113-Glu119). The structure includes numerous hydrophobic cavities that may contribute to ligand binding. Assessment of the structure and superimposition of the model with the template revealed that the six α-helices superimposed with an RMSD value of 0.386 Å, indicating identical folds for MsepCSP8 and the template structure (**Figure 6D**).

Furthermore, molecular docking was performed to explore the mechanism and binding affinity of ligands to MsepCSP8. Interactions between binding sites of MsepCSP8 and functional residues of ligands are shown in **Table 2**. The docking simulation indicated that Glu-122 and Lys-121 were the main residues participating in ligand binding, and several other residues also interacted closely with ligands.

**Table 2:** Molecular docking results of tested ligands.

PubChem ID	Ligand	*S*-Score	RMSD value	Residue interacting with H-bonding	Closer contact interacting residues
6184	Hexanal	-19.90	1.08	–	Ile-92, Trp-99, Lys-100, Gly-110, Glu-117, Lys-121, Glu-122, Val-123
11463	Terpinolene	-18.09	0.95	–	Lys-100, Cys-103, Gly-110, Ser-114, Glu-117, Lys-121, Glu-122, Val-123
11622	2-Tridecanone	-17.88	1.35	–	Lys-100, Cys-103, Ala-104, Gly-110, Ser-114, Glu-117, Lys-121, Glu-122, Val-123
7462	α-Terpinene	-17.54	0.49	–	Cys-103, Gly-110, Ser-114, Glu-117, Lys-118, Lys-121, Glu-122, Val-123
12020	1-Penten-3-ol	-17.68	0.36	Glu-122	Ile-92, Lys-100, Cys-103, Glu-117, Lys-121, Val-123
7966	Cyclohexanol	-17.70	0.54	Glu-122	Ile-92, Cys-103, Glu-117, Lys-121, Val-123
454	Octanal	-17.98	0.65	–	Ile-92, Pro-96, Trp-99, Lys-100, Cys-103, Gly-110, Ser-114, Glu-117, Lys-121, Glu-122, Val-123
5281168	*Trans*-2-hexenal	-16.57	1.75	–	Lys-100, Cys-103, Gly-110, Glu-117, Lys-121, Glu-122, Val-123
11230	(–)-Terpinen-4-ol	-16.70	1.65	Lys-121	Ile-92, Cys-103, Gly110, Ser-114, Glu-122, Val-123

*RMSD, root mean square deviation.*

**FIGURE 7:**
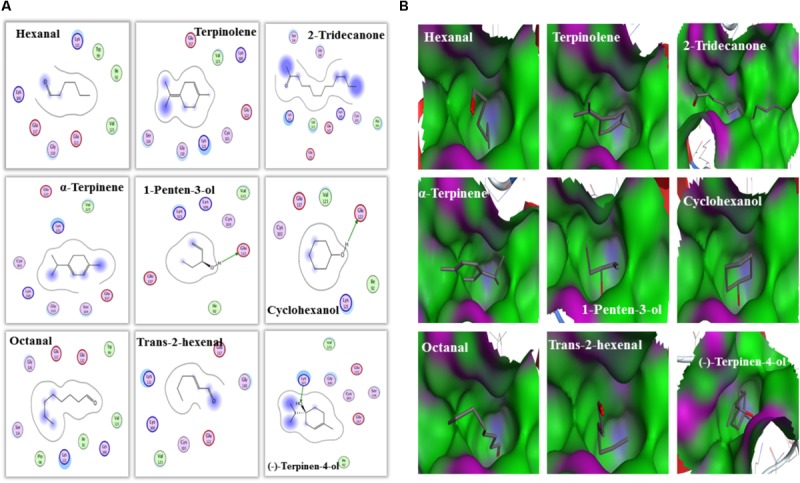
Amino acid residues and bind cavities of MsepCSP8. **(A)** Two dimensional interaction view of MsepCSP8 amino acid residues. The red and green amino acids indicate polar and non-polar, respectively. Arrows with dashed lines represent hydrogen-bonding. **(B)** Binding cavities of MsepCSP8. The red and green area denote hydrophilia and hydrophobicity, respectively. The red atoms show oxygen atoms.

An interaction model of potential residues of MsepCSP8 interacting with ligands is shown in **Figure 7A**. Six amino acid residues interact with 1-penten-3-ol, five residues interact with cyclohexanol. Glu-122 forms a hydrogen bond with these two ligands. Likewise, six amino acid residues may interact with (-)-terpinen-4-ol, and Lys-121 may engage in H-bonding with this ligand. Other ligands also appeared to interact with various amino acid residues. Molecular docking also revealed that the tested ligands could bind strongly the center of the MsepCSP8 pocket, and thereby influence its activity. Furthermore, some ligands may bind to a tunnel in the core of the MsepCSP8 structure. The nine selected ligands may dock at the same binding sites, and all interactions with ligands appeared to involve residues from the six α-helices (**Figure 7B**).

### Behavioral Responses of *M. separata* to Ligands Displaying High Binding Affinity to MsepCSP8

**FIGURE 8:**
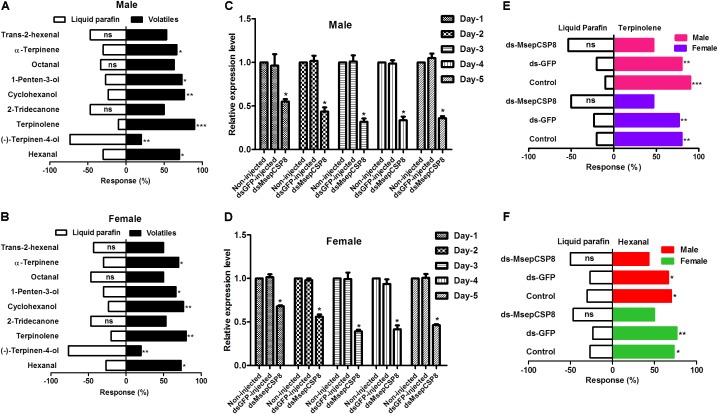
Effects of dsRNA injection on the behavioral responses to volatiles of *M. separata*. **(A)** Male without injection of dsRNA; **(B)** females without injection of dsRNA. **(C,D)** Effects of dsRNA injection on MsepCSP8 mRNA level in male and female. **(E,F)** Behavioral response of *M. separata* to terpinolene and hexanal after injection of dsRNA. Control, adult without injection of dsRNA; dsGFP, adult injected with dsRNA of green fluorescent protein; dsMsepCSP8, adult injected with dsRNA of MsepCSP8. ^∗^*p* ≤ 0.05, ^∗∗^*p* ≤ 0.01, ^∗∗∗^*p* ≤ 0.001, respectively.

Nine volatiles that bound strongly (Ki <10 μM) to MsepCSP8 in competitive binding assays were chosen to study the behavioral responses of male and female insects (**Figures 8A,B**). Males and females exhibited similar trends in response to the tested volatiles; both displayed a distinct preference (*p* ≤ 0.05) for alcohols (cyclohexanol and 1-penten-3-ol). In the case of aldehyde volatiles, hexanal induced a significantly higher response (males = 70%, *p* = 0.0285; females = 73.33%, *p* = 0.0106), whereas octanal and *trans*-2-hexenal induced non-significant response. The ketone volatile (2-tridecanone) also elicited non-significant attraction in both male and female moths. *M. separata* individuals exhibited varied patterns in response to monoterpenes; moths were highly attracted to terpinolene (males = 90%, female = 80%) and α-terpinene (males = 66%, females = 70%) but repelled by (–)-terpinen-4-ol.

### Effectiveness of RNAi on MsepCSP8

Expression of MsepCSP8 was suppressed in dsRNA-treated insects, and this suppression increased over time until the 3rd day post-eclosion in both sexes (**Figures 8C,D**). After dsMsepCSP8 injection, olfactometer bioassays were applied to examine the efficiency of knockdown on the behavior of males and females at 3 days post-eclosion. Two compounds (terpinolene and hexanal) that elicited the strongest attractive responses before RNAi were selected, and after dsMsepCSP8 injection, the behavioral responses of adults were not significantly affect by either of the two compounds (**Figures 8E,F**).

## Discussion

Analysis of the expression of CSPs can provide clues to gene function in insects. Herein, levels of MsepCSP8 transcripts in *M. separata* adults were significantly higher than those in larvae and pupae, indicating potential roles in host or mate location. Further analysis of body tissues revealed highest expression in levels in antennae, suggesting it may be functionally involved in chemodetection. Antennae are the main olfactory organ of insects and they sense a large number of volatiles with numerous functions (Zhang Y.N. et al., 2013). Several previous studies reported that genes encoding CSPs are highly expressed in antennae (Calvello et al., 2005; Zhang and Lei, 2015). MsepCSP8 expression in wings and legs may indicate gustatory and contact functions, as well as a role in behavioral adaptation (He et al., 2011; Hua et al., 2013). Gong et al. (2007) found that expression of BmorCSP10 in *Bombyx mori* was notably higher in contact organs (antennae, wings, and legs) than in non-contact organs (head, thorax, and abdomen), suggesting it is important for contact chemoreception. Expression of MsepCSP8 in the abdomen of both sexes was negligible, suggesting that this gene may play only a minor role in specific chemosensing during mating or oviposition (Hua et al., 2013). In addition, in all body parts, sex-based expression of MsepCSP8 was consistently higher in females than males, implying a sex-based function. The broad spectrum of MsepCSP8 expression in *M. separata* indicates that CSP genes might have other functions apart from chemosensation (Guo et al., 2011, Gu et al., 2012; Zhang et al., 2012).

Analyses revealed that pH substantially influenced the binding affinity of ligands to MsepCSP8; most ligands (20 out of 56) bound more tightly (Ki < 10) at pH 7.4 than at pH 5.0, while 11 bound more strongly at pH 5. In the case of alcoholic compounds, cyclohexanol, 1-penten-3-ol, 1-octen-3-ol and *trans*-2-hexen-1-ol all bound strongly to MsepCSP8. The strong binding affinity of ligands with CSP is associated with location and recognition of hosts (Zhang and Lei, 2015). Sun et al. (2014) showed that HoblCSP2 has a high affinity for alcoholic compounds, and Yin et al. (2012) found that the alcoholic ligand *cis*-3-hexen-1-ol binds strongly to LstiGOBP2 of *Loxostege sticticalis*. Most of the tested compounds with an aldehyde group (*trans*-2-hexenal, hexanal, octanal, nonanal, and 2-methylbutyraldehyde) also displayed high affinities for MsepCSP8. Ban et al. (2003) demonstrated that CSPs from *S. gregaria* bind strongly to cinnamaldehyde and 2-amylcinnamaldehyde. This specific binding to aldehyde groups suggests that MsepCSP8 might have an appropriate binding site for this functional group, indicating an important role in chemoreception in *M. separata*. Zhang T.T. et al. (2013) also revealed that aldehyde compounds bind strongly to CSPs, confirming their vital roles in chemoreception in cotton bollworm. Monoterpenes (–)-terpinen-4-ol, (R)-(+)-β-pinene, (S)-(–)-verbenone, (+)-3-carene, α-terpinene and terpinolene, and the sesquiterpene (+)-α-longipinene displayed high binding affinities toward MsepCSP8. He et al. (2011) also revealed extremely high binding affinities for terpenes to NlugOBP3, while Ming and Peng (2014) reported that among terpenes, nerolidol has high affinity whereas limonene and α-pinene exhibit moderate binding affinity to SfurOBP2, suggesting these volatiles might be attractants or deterrents in *Sogatella furcifera* and other insects. The ligands with ketone group, 2-tridecanone and β-ionone also exhibited a high affinity for MsepCSP8. He et al. (2011) investigated the binding affinity of rice plant volatiles (2-tridecanone and β-ionone) and demonstrated high binding affinity to NlugOBPs from *Nilaparvata lugens*. Only one compound with an ester and benzoate group (methyl benzoate) and two compounds with an alkane group (hexane and n-heneicosane) bound highly to MsepCSP8, suggesting MsepCSP8 may be involved in detecting various host volatiles (Yin et al., 2012). MsepCSP8 displayed strong affinity toward wide variety of compounds, indicating involvement in the olfactory system of *M. separata*. MsepCSP8 also displayed medium and weak binding to some of the tested volatiles, suggesting it may function in the recognition and transport of these volatiles.

To further validate the results of ligand binding, 3D modeling and molecular docking of ligands were performed. The predicted 3D structure of MsepCSP8 has the typical features of CSPs, with six α-helices and an internal cavity (Zhang et al., 2012). The hydrophobic binding pocket of MsepCSP8 is similar to that of CSPsg4 from *S. gregaria* and CSPMbraA6 from *M. brassicae* (Tomaselli et al., 2006). Ligand binding depends on the specific amino acids positioned in this hydrophobic region (Tian et al., 2016). For instance, Tyr26 is essential for 12-bromo-dodecanol binding to CSPMbraA6 (Campanacci et al., 2003). In the present study, several residues including Ile-92, Trp-99, Lys-100, Gly-110, Glu-117, Lys-121, Glu-122, Val-123, Cys-103, Ser-114, Ala-104, Lys-118, Pro-96, and Trp-99 might be essential for ligand binding in MsepCSP8. Furthermore, hydrogen bonding between residues (Glu-122 and lys-121) and ligands suggests that some amino acid residues are vital in the interaction of MsepCSP8 with volatile compounds.

To further support the binding assay results, the behavioral responses of *M. separata* to volatiles were monitored. The responses of both sexes to alcoholic compounds (cyclohexanol and 1-penten-3-ol) were positive, indicating attraction of moths toward plants releasing these volatiles. The attraction of *M. separata* to alcoholic compounds corresponds with the results of previous studies that reported positive responses of several other insect species to alcoholic compounds (Light and Jang, 1987; Ramachandran et al., 1990; Das et al., 2013; Cao et al., 2015; Lihuang et al., 2017). Lihuang et al. (2017) found that male and female *M. separata* individuals presented similar responses to alcoholic compounds. It is plausible that compounds tested in our study could play an important role in locating host plants. Among the tested aldehyde compounds, hexanal attracted both males and females, whereas 2-tridecanone failed to attract both sexes. The identification of semiochemicals attracting or repelling specific insects may enable the development of insect pest management strategies (Das et al., 2013).

In the present study, a noticeable reduction in dsMsepCSP8 mRNA levels was achieved by microinjection. The failure of adults displaying a preference for volatiles after RNAi treatment implies that disruption of olfaction caused by dsRNA prevented adults from detecting odors. It has been reported that dsRNA treatment of *Aenasius bambawalei* also inhibited responses to volatiles (Li et al., 2018), and *Dastarcus helophoroides* was similarly non-responsive to volatiles after dsRNA injection (Yang et al., 2017).

## Conclusion

The present study assessed the function MsepCSP8 in *M. separata* and demonstrated a plausible involvement in chemoreception, in addition to other functions. Fluorescence binding bioassays, structural modeling, molecular docking and behavioral responses further validated these proposed functions. Furthermore, the reduction in MsepCSP8 transcripts after dsMsepCSP8 injection decreased the behavioral responses of *M. separata* following exposure to attractive volatiles. Thus, MsepCSP8 might play key roles in chemoreception and other physiological functions in *M. separata*.

## Author Contributions

AY and M-QW conceived and designed the experiments. AY performed the experiments. AY, MW, MTuQ, MS, and M-QW analyzed the data. AY, SP and M-QW wrote and edited the manuscript.

## Conflict of Interest Statement

The authors declare that the research was conducted in the absence of any commercial or financial relationships that could be construed as a potential conflict of interest.

## Footnotes

1 http://www.ncbi.nlm.nih.gov/

2 http://www.ncbi.nlm.nih.gov/tools/primer-blast/

3 http://blast.ncbi.nlm.nih.gov/Blast.cgi

4 http://www.rcsb.org

5 http://swissmodel.expasy.org/

6 http://web.expasy.org/compute_pi/

## Supplementary Material

FIGURE S1 Phylogenetic tree of MsepCSP8 with 114 CSPs sequences from other insect species.

FIGURE S2 The Ramachandran plot of MsepCSP8.

TABLE S1 Primers used in present study.
