# Supplementary figures and images for: Functional Analysis of the Chemosensory Protein MsepCSP8 From the Oriental Armyworm Mythimna separata
Source: Front Physiol. 2018 Jul 12;9:872. doi: 10.3389/fphys.2018.00872 (PMC6052345; doi:10.3389/fphys.2018.00872)

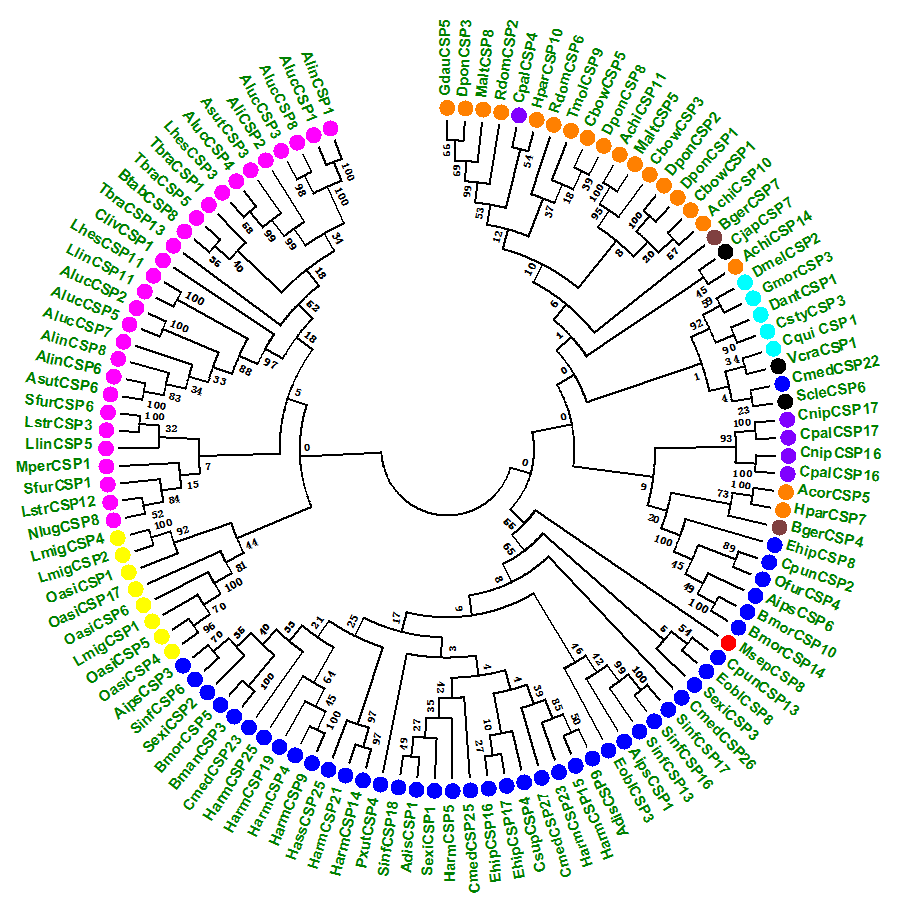

Supplement: FIGURE S1 — Phylogenetic tree of MsepCSP8 with 114 CSPs sequences from other insect species. [file Image_1.png]

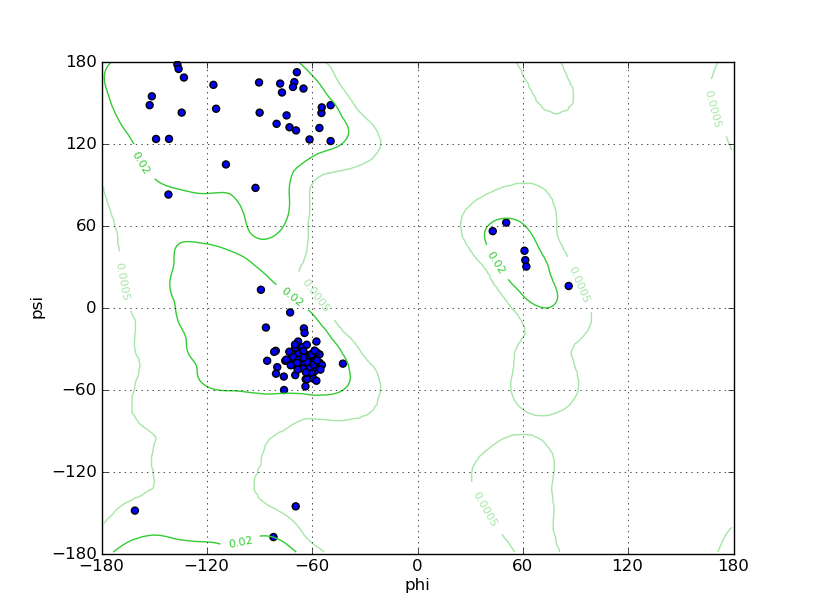

Supplement: FIGURE S2 — The Ramachandran plot of MsepCSP8. [file Image_2.png]
