# Supplementary material for: Functional Analysis of the Chemosensory Protein MsepCSP8 From the Oriental Armyworm Mythimna separata
Source: Front Physiol. 2018 Jul 12;9:872. doi: 10.3389/fphys.2018.00872 (PMC6052345; doi:10.3389/fphys.2018.00872)
Supplement: TABLE S1 — Primers used in present study. [file Table_1.DOCX]

**Table S1.** Primers used in present study.

|  | **Primer names** | **Primer sequences (5´to 3´)** |
| --- | --- | --- |
| **For RT-qPCR** | MsepCSP8-F | AGTGCTCGCCTAAGCAGAAA |
|  | MsepCSP8-R | TTGCCTTCGGGGTCGTATTT |
|  | β-Actin-F | AGATCAAGATCATCGCGCCC |
|  | β-Actin-R | GGAAGGTCCGCATTCATCGT |
| **For dsRNA synthesis** | MsepCSP8-F | TAATACGACTCACTATAGGATGAAAACCTTATTCATTCTGTGT |
|  | MsepCSP8-R | TAATACGACTCACTATAGGTTATTGAGAGACTTCTTTGAGTTCT |
|  | GFP-F | TAATACGACTCACTATAGGAAGGGCGAGGAGCTGTTCACCG |
|  | GFP-R | TAATACGACTCACTATAGGCAGCAGGACCATGTGATCGCGC |
